# Role of Ultrasonography in Monitoring Chemotherapeutic Effects on Primary Thyroid Lymphoma: A Single-Center Retrospective Study

**DOI:** 10.3390/medicina61010015

**Published:** 2024-12-26

**Authors:** Shirong Liu, Ying Fu, Ligang Cui, Shumin Wang, Shi Tan

**Affiliations:** Department of Ultrasound, Peking University Third Hospital, Beijing 100191, China

**Keywords:** lymphoma, thyroid neoplasms, doppler, ultrasonography

## Abstract

*Background and Objectives:* In this study, we assessed the utility of ultrasonography in monitoring the chemotherapeutic effects on primary thyroid lymphoma (PTL). *Materials and Methods:* This retrospective analysis included 17 patients with PTL who received chemotherapy from 2012 to 2022. The sonographic features were examined pre- and post-treatment using ultrasound (US) to monitor the treatment response at the first to second, third to fourth, and end cycles of chemotherapy and follow-up, and progression-free survival (PFS) and overall survival (OS) were analyzed. *Results:* The sonographic findings for all the patients indicated diffuse or nodular infiltration with markedly hypoechoic masses, and “stripe-shaped” high echoes and posterior acoustic enhancement were observed. Following one to two cycles of chemotherapy, a US examination revealed varying tumor reduction degrees and diminished blood flow signals. After three to four cycles of chemotherapy, the US demonstrated an evaluation efficacy comparable to that of PET-CT in cases in which the lesion had entirely disappeared postchemotherapy; however, its ability to differentiate between treatment response and residual lesions was less effective compared to that of PET-CT. After the end cycle of chemotherapy, the lesion sizes had significantly decreased compared to those at the baseline (*p* < 0.05). Postchemotherapy, Adler’s blood flow grades decreased significantly, with 80% graded as 0–1. Among the 10 patients with cervical lymph node enlargement, 70% showed reduced lesion sizes and blood flow signals. The cumulative 5-year PFS and OS rates were both 80% for the diffuse type and 82.5% and 78.8% for the nodular type, respectively (*p* > 0.05). *Conclusions:* US can be utilized to monitor the therapeutic response following chemotherapy for PTL, especially for early assessment and repeated dynamic monitoring, and can serve as a complementary follow-up method to PET-CT.

## 1. Introduction

Primary thyroid lymphoma (PTL) is rare, comprising 1–5% of all thyroid gland malignancies, 1–2% of all extranodal lymphomas, and 3% of all non-Hodgkin lymphomas. PTL predominantly affects older women with a history of Hashimoto’s thyroiditis [1] and usually has a good prognosis; however, it can exhibit sudden and rapid growth, resembling undifferentiated cancer, and can be life-threatening due to the narrowing of the trachea or asphyxia owing to tumor enlargement. Therefore, early diagnosis and treatment are imperative for optimizing the prognosis and initiating timely interventions. Surgery plays a limited role in PTL management, as it does not enhance the prognosis but may aid in tissue diagnosis or occasionally alleviate severe airway obstruction [2]. The primary approach to PTL treatment involves chemotherapy, often in conjunction with radiotherapy. Traditional imaging modalities, such as computed tomography (CT) and magnetic resonance imaging (MRI), are primarily limited to providing information on morphological alterations and cannot be used to assess metabolic activity. Furthermore, CT involves exposure to ionizing radiation, while MRI is predominantly utilized for evaluating lymphoma affecting the central nervous system. Positron emission tomography-computed tomography (PET-CT) is important for assessing the lymphoma treatment efficacy; however, its drawbacks include the cost, radiation exposure, and false positives attributed to inflammation and tumor necrosis [3]. Ultrasonography, distinguished by its noninvasive nature, convenience, and excellent repeatability, offers several advantages and can be used to assess the mass size, internal echogenic changes, internal blood flow, and surrounding lymph nodes. Although prior reports on the sonographic features of PTL exist [4], these studies are limited in terms of their sample sizes and rarely report any postchemotherapy sonogram changes. This retrospective analysis encompassed 17 patients with PTL diagnoses who were treated with standard chemotherapy at our hospital over the past decade to elucidate whether ultrasonography can be effectively used to monitor the chemotherapeutic effect on PTL.

## 2. Materials and Methods

### 2.1. Study Design and Patients

This single-center, retrospective study included data from 17 patients who were diagnosed with and treated for PTL at our institution between January 2012 and December 2022. The patient cohort consisted of two males and fifteen females, aged 34–88 years (mean: 66.3 ± 20.7; median: 67 years; Table 1). All the patients possessed comprehensive medical records and underwent standardized chemotherapy. Gray-scale and color Doppler ultrasound (CDU) were employed pre- and post-treatment. Additionally, one patient underwent a contrast-enhanced ultrasound (CEUS) examination. The exclusion criteria encompassed incomplete data, the unavailability of ultrasound (US) data within our hospital, patients with contraindications to chemotherapy, and an expected survival of less than 2 months. This study was approved by our hospital Ethics Committee (Ethical Project Number LM2023557; 7 September 2023), and the need for obtaining written informed consent was waived due to its retrospective nature.

### 2.2. Sonographic Examinations

US examinations were conducted using a GE LOGIQ E9 (GE Healthcare, Wauwatosa, WI, USA), an HI VISION Avius L (Hitachi High-Tech Co., Tokyo, Japan), and a Samsung RS80A (Samsung Medison Co. Ltd., Seoul, Republic of Korea) fitted with a high-frequency linear array probe. A low-frequency convex array probe was employed for larger masses. Thyroid and cervical lymph nodes were scanned on transverse, longitudinal, and multiple oblique planes. The clinical parameters and sonographic data were retrieved from electronic medical records and were reviewed by two authors, each with >5 years of corresponding experience. The recorded data included the thyroid size, background echotexture, and ultrasonic characteristics. Sonography PTL appearances were categorized into three types: diffuse, nodular, and mixed [5]. Lesions were measured in three axes for the nodular types, and the volume (V) was calculated using the formula V = 0.52abc, where a, b, and c represent the length, width, and thickness of the nodule, respectively. The thyroid gland thickness was measured for the diffuse and mixed types. The internal blood flow supply was graded into four categories using the Adler semi-quantitative method [6].

### 2.3. Treatment Protocol, Efficacy Evaluation, and Follow-Up

All patients underwent chemotherapy, receiving R-CHOP (rituximab, cyclophosphamide, doxorubicin, vincristine, and prednisone) or a CHOP-like regimen. The exception was one case of mucosa-associated lymphatic tissue lymphoma, which received the R-CVP regimen (rituximab combined with cyclophosphamide, vincristine, and prednisone). The duration of all chemotherapy cycles ranged from 2 to 3 weeks. If the lesion persisted after four treatment cycles, then a recommendation was made to modify the chemotherapy regimen. The imaging modalities included US and PET-CT. Gray-scale US and CDU were performed concurrently following the first to second, third to fourth, and end cycles of the treatment regimen. The ultrasonic monitoring standard includes the evaluation of the lesion size, along with an assessment of the Alder blood flow signal grade. PET-CT follow-up scans are scheduled after the third to fourth and end cycles. The treatment response was evaluated using the Lugano 2014 evaluation criteria [7], categorizing the outcomes into four types: complete remission (CR), partial remission (PR), stable disease (SD), and disease progression (PD). CR and PR were considered the overall response (OR), while SD and PD were considered no response (NR). A comparative analysis was conducted to evaluate the efficacy of the US and PET-CT in assessing the treatment response after three to four cycles of the treatment. The follow-up included outpatient visits and telephone communication up to 31 December 2022, or the date of death. Progression-free survival (PFS) was defined as the interval from the initiation of therapy to tumor progression, encompassing local tumor progression or lesions involving other organs or patient death. Overall survival (OS) was calculated from the start of the initial therapy to the patient’s death from any cause or the last known contact.

### 2.4. Statistical Analyses

The data analysis was performed using SPSS (Version 17.0; IBM, Armonk, NY, USA). Baseline data are presented as means ± standard deviations for the quantitative variables and as percentages for the qualitative variables. The mean quantitative values were compared using Student’s *t*-test, nonparametric data were assessed using the Mann–Whitney U test, and the proportional differences were analyzed using the chi-square test. The Kaplan–Meier method was used to estimate PFS and OS, and the log-rank test was used to compare the differences. A *p*-value < 0.05 was considered statistically significant.

## 3. Results

### 3.1. Pretreatment Ultrasonographic Findings

The lesions exhibited the diffuse type in five cases (5/17, 29.4%) and the nodular type in twelve cases (12/17, 70.6%). Among the five diffuse-type cases, all displayed varying degrees of asymmetric thyroid enlargement, some with unilateral lobe enlargement (3/5, 60%). Of the 12 patients with the nodular type, 10 cases (83.3%) presented with single nodules, while two cases (16.7%) had multiple nodules, totaling 15 (Table 1). The echogenic characteristics of the two types are shown in Table 2.

### 3.2. Treatment Response

**Figure 1:**
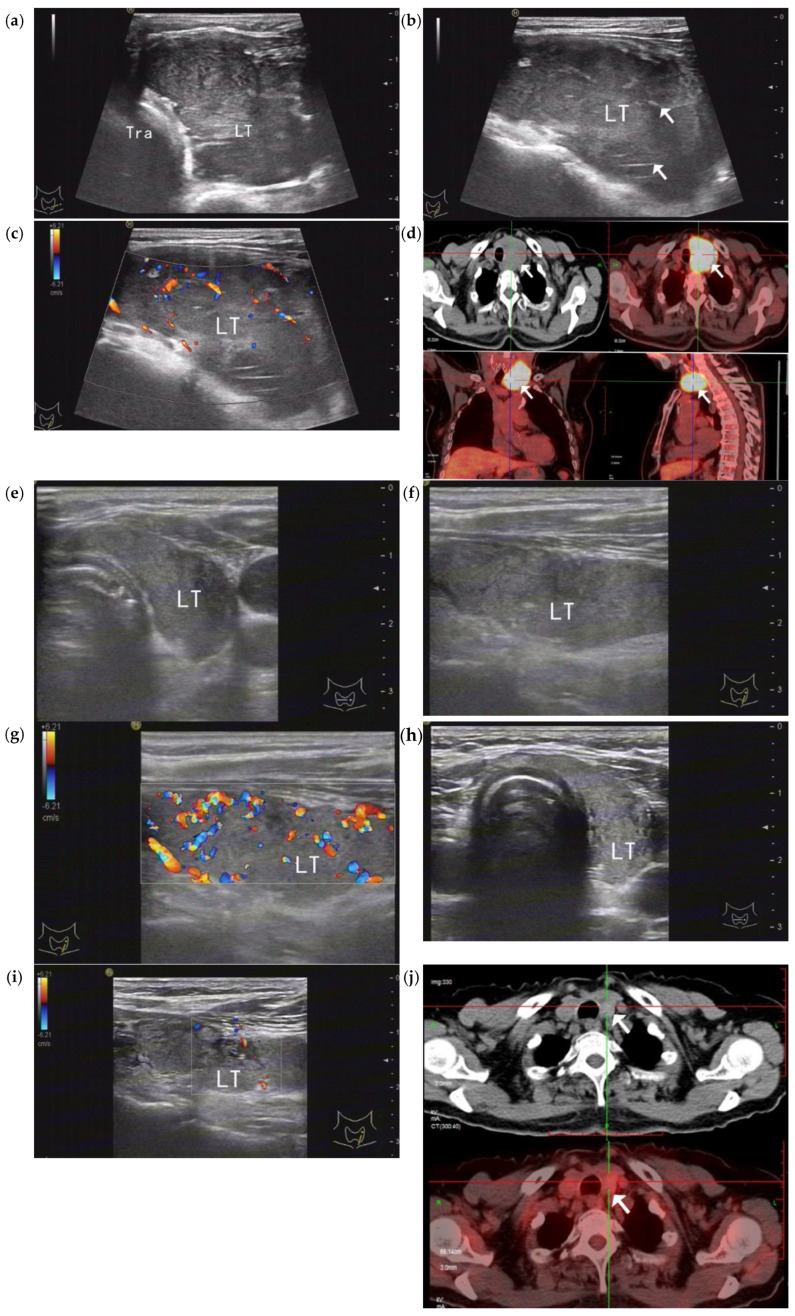
An elderly woman with diffuse--type PTL (DLBCL subtype) in the LT who underwent a right thyroidectomy 17 years ago due to Hashimoto’s thyroiditis. Regular follow-ups with ultrasound indicated a significant enlargement of the LT. (**a**,**b**) Gray-scale ultrasound of the transverse and longitudinal sections before chemotherapy shows a markedly increased LT volume, with a maximum thickness of 3.6 cm and internal “stripe-shaped” high echoes (arrows). (**c**) CDU showing abundant blood flow signals. (**d**) PET-CT before chemotherapy showing significant enlargement of the LT and an abnormal radiation uptake increase (arrows), with an SUVmax of 25.9. (**e**–**g**) After two cycles of the R-CHOP regimen, the LT thickness significantly decreased to 1.7 cm, and the parenchymal echo increased but still showed rich blood flow signals. Considering the treatment’s effectiveness, continuing the original chemotherapy regimen was recommended. (**h**,**i**) After four cycles of chemotherapy, the ultrasound showed a normal thyroid thickness, an increased glandular echo, which had returned to normal, the disappearance of the lesion, and a decreased blood flow signal. (**j**) PET-CT confirmed no metabolic increase in the lesion, with an SUVmax of 2.0 (arrows). PTL, primary thyroid lymphoma; DLBCL, diffuse large B-cell lymphoma; LT, left lobe; CDU, color Doppler ultrasound; PET-CT, positron emission tomography-computed tomography; SUVmax, maximum standardized uptake value; R-CHOP, rituximab, cyclophosphamide, doxorubicin, vincristine, and prednisone.

In the diffuse-type group, all five patients demonstrated varying degrees of remission on the US examination after one to two cycles of therapy, characterized by decreases in the thyroid gland thickness and blood flow signal. Following three to four cycles of chemotherapy, the overall CR rate was 80% (4/5), and 60% (3/5) showed the complete resolution of lesions, confirmed by both US and PET-CT imaging (Table 3; Figure 1). Another patient’s US images revealed that the thyroid gland thickness had returned to normal; however, a small patch-like hypoechoic area measuring 0.5 × 0.4 × 0.3 cm was identified in the left lobe, which exhibited mild elevated metabolic activity on the subsequent PET-CT (standardized uptake value (SUV): 4.0). The final US-guided biopsy confirmed this as a post-treatment reaction rather than a residual tumor, also indicating the CR status. A PD rate of 20% (one-fifth) was observed after nine cycles of chemotherapy. This patient initially showed PR after four cycles of chemotherapy, with the left lobe thickness decreasing from 4.0 to 1.7 cm in the US images. However, a small amount of residual tissue (1.6 × 1.3 × 1.0 cm) in the lower part was subsequently confirmed via PET-CT (SUV: 7.4) and a biopsy. Tumor progression was identified nine cycles after the initial therapy, and despite changing the regimen, the tumor continued to progress. The patient eventually died of a severe lung infection, with a total survival duration of 12 months.

Regarding the nodular-type group, 86.7% (13/15) of the nodules demonstrated varying degrees of remission on the US examination after one to two cycles of therapy, and 13.3% (2/15) showed nodule size increases. The overall CR rates after the third to fourth and end cycles of treatment were 20% (3/15) and 66.7% (10/15), respectively. The PR, SD, and PD rates after three to four cycles were 53.3% (8/15), 13.3% (2/15), and 13.3% (2/15), respectively, and the rates at the end cycles were 13.3% (2/15), 13.3% (2/15), and 6.7% (1/15), respectively—i.e., after three to nine cycles of chemotherapy (Table 3). Among the ten nodules that eventually achieved a CR, eight (8/10, 80%) were completely resolved on both the US and PET-CT images. One nodule demonstrated a CR on PET-CT after five cycles of chemotherapy; however, it appeared as a well-defined hypoechoic area measuring 2.2 × 0.4 cm with a grade 1 blood flow signal on the US images. The PET-CT findings were confirmed as a post-treatment reaction, with the SUV decreasing from 32.0 to 1.1 (Figure S1). Another nodule demonstrated PR after seven cycles of treatment; however, the subsequent US follow-up indicated an enlargement of the lesion, suggesting recurrence. Nevertheless, a CR was achieved after modifying the treatment regimen. One PD nodule that was initially evident exhibited shrinkage, with a focal hyperechoic area measuring 0.5 × 0.4 cm after two cycles of therapy, as observed on the US images. Notably, this nodule underwent multiple CEUS examinations both before and after the treatment. The pretreatment CEUS revealed the rapid and homogeneous enhancement of the lesion, with no non-enhancing areas observed. By the subsequent CEUS follow-up, the lesion had gradually enlarged, accompanied by an increase in the blood flow perfusion, as evidenced by the increased number of contrast agent microbubbles and the higher peak intensity (PI) on the time–intensity curve (TIC). Despite altering the treatment regimen, the nodule continued to enlarge, and the patient died of heart failure 14 months after the initial therapy.

Following the end cycles of the chemotherapy regimen, the lesion size, including the thyroid gland thickness or maximum diameter and calculated volume of the nodules, significantly decreased in all 17 patients compared with those before chemotherapy (*p* < 0.05; Table 4). Additionally, the Adler blood flow grading demonstrated a marked decrease post-therapy, with 80% of the lesions classified as grades 0–1 (Table 5). Concerning the 10 patients with cervical lymph node enlargement, the post-treatment outcomes revealed CR in seven cases, SD in two cases, and PD in one case.

Among the seventeen patients, three experienced recurrences during the follow-up period, and two achieved a CR after adjusting the chemotherapy regimen. The cumulative PFS rates for the diffuse type at 1 year and 5 years were both 80%, showing no significant difference compared to the nodular type (91.7% and 82.5%, respectively) (*p* = 0.985). There were three deaths among the 17 patients, all belonging to subtypes of diffuse large B-cell lymphoma. The causes of death included lymphoma, severe pulmonary infection, and heart failure. The 1-year and 5-year cumulative OS rates for the diffuse type were both 80%, while those for the nodular type were 90% and 78.8%, respectively. No statistically significant difference was observed between the two groups (*p* = 0.932) (Figure S2).

## 4. Discussion

Treating PTL primarily relies on systemic approaches, such as chemotherapy, which alone has yielded a CR rate of 77–100%, with the addition of rituximab significantly improving the OS rates of patients with PTL [1]. This report is consistent with the results of this study; however, some patients do not respond positively and may even experience rapid progression after common chemotherapy regimens, impacting their survival. Therefore, the accurate and timely evaluation of the effectiveness of chemotherapy is crucial for improving outcomes. Recent advancements in high-frequency US have enhanced the instrument resolution, allowing for the clear visualization of fine tumor structures. We conducted a US examination to assess the impact of chemotherapy on 17 patients with PTL. To the best of our knowledge, there are limited reports on this topic. The advantages of this technique are as follows. First, US not only elucidates the morphological characteristics of tumors but also provides real-time blood flow and peripheral lymph node information, rendering it an efficacious imaging technique for assessing the chemotherapy effectiveness in PTL. Second, the US examination is characterized by its noninvasiveness, convenience, and good reproducibility, enabling it to dynamically and promptly monitor the tumor’s response to chemotherapy during the early stages (i.e., after one to two cycles), and it serves as a valuable supplementary tool to PET-CT for monitoring the treatment efficacy, facilitating the timely adjustment of chemotherapy regimens. Third, the absence of ionizing radiation in US facilitates repeated examinations, thereby mitigating the risk of excessive radiation exposure associated with multiple PET-CT scans.

In this study, the patients with PTL in the OR group showed significant reductions in or the complete resolution of the thyroid thickness and nodule size and volume compared with the pretreatment levels. In contrast, the lesions in the non-OR group exhibited no significant changes, or they increased, reflecting the inhibitory effect of chemotherapy drugs on the malignant proliferation rate of lymphocytes in the thyroid. Our results reveal that US enables the continuous and repetitive observation of the size changes in PTL lesions postchemotherapy. However, simply reflecting morphological changes is not enough. When a patient responds well to chemotherapy, the tumor metabolism changes before a visible decrease in size can be detected, which leads to a false-negative result in the treatment response assessment.

The findings of our study indicate that US and PET-CT exhibit comparable efficacies in assessing the complete disappearance of lesions following chemotherapy; however, US is less effective than PET-CT at distinguishing between post-treatment responses and residual lesions, and the possible reasons are as follows: CDU can detect blood flow signals within tumors, thereby providing insights into their metabolic information to some extent. However, owing to the susceptibility of CDU to the influence of the blood flow direction and detection angle during the imaging process, it may not yield satisfactory results for small or low-flow-velocity vessels, potentially failing to accurately reflect the true biology of tumor angiogenesis. Several studies have reported significant variability in the blood flow signals of PTL, with high vascular proliferation being the most prevalent feature [8]. This phenomenon may be associated with the greater aggressiveness of DLBCL and the increased density of the surrounding blood vessels, which aligns with the results of this study, which indicate that 95% of the pretreatment blood flow signals were classified as grades 2–3. In this study, Adler grades 3 and 2 were evident in 65% and 30% of the patients, respectively, and significantly decreased post-treatment, with grades 0–1 accounting for 80% of the patients. This suggests a potential correlation with the direct action of chemotherapy drugs on endothelial cells, leading to tumor blood vessel thinning or occlusion, reflecting the chemotherapy effectiveness.

The CEUS results for one patient in our study suggest a potential pretreatment PTL blood flow pattern characterized by rapid and uniform enhancement without any non-enhancing area. This observation is consistent with the typical CEUS features of lymphoma as described by Rubaltelli et al. [9], which include a diffuse distribution of “snowflake”-like spot enhancement, which subsequently coalesces into a homogeneous enhancement pattern. We hypothesize that this phenomenon may be attributed to the highly vascularized nature of lymphoma, featuring enlarged and thickened vessels originating from the portal and perivascular regions [10], which facilitate the rapid diffusion of CEUS contrast agent microbubbles throughout the lesion upon entry, thereby reducing the likelihood of perfusion defects. Previous studies have indicated that CEUS can be employed to assess tumor perfusion [11] and may serve as a valuable tool for assessing the treatment response in patients with lymph node lymphomas [12]. Our results suggest that CEUS may be useful in distinguishing between a residual tumor and post-treatment response in cases in which there is no change in the size during multiple follow-up examinations. We speculate that residual lymphoma lesions may present as high enhancement. In contrast, the post-treatment response may appear as uniform hypoperfusion or consistent with the surrounding thyroid parenchyma, indicating fibrosis or Hashimoto’s thyroiditis post-treatment; however, only one case underwent CEUS. As the treatment progresses, CEUS enables real-time monitoring of the size and morphological alterations of lymphoma lesions. However, its particular strength lies in its observation of changes in the blood flow perfusion, which can be quantitatively assessed through the TIC curve. This approach potentially offers enhanced sensitivity over CDU in evaluating the tumor’s response to chemotherapy. Consequently, under efficacious treatment, the influx of microbubbles into the PTL lesion is expected to diminish, leading to a reduction in the peak intensity (PI), an extension of the time to peak (TTP), or a shortening of the washout time. Possible explanations for these changes include the potential for chemotherapy to impair the vascular architecture within the tumor, thereby reducing the vascular density and slowing the blood flow. Nonetheless, there is a paucity of data regarding the use of CEUS for monitoring the chemotherapy response in PTL, and only one case in this study was evaluated both pre- and post-treatment. Thus, additional research is warranted to validate these findings.

Our results underscore the crucial role of US in the early evaluation of the PTL treatment efficacy. In this study, US assessments were conducted within one to two cycles of treatment to facilitate timely adjustments to the treatment regimen if the initial therapy was deemed ineffective. This approach addresses the limitations of traditional imaging modalities (such as CT and PET-CT) in assessing lymphoma treatment efficacy, in which follow-up evaluations are typically scheduled after three cycles of chemotherapy. Such a delayed assessment can result in overlooking the rapid morphological and functional changes in lymphoma lesions during the early stages of treatment [13], thereby hindering the timely modification of the treatment plan.

Despite these promising results, this study had some limitations. First, this study was conducted at a single center with a small sample size, and it had a retrospective design, introducing methodological weaknesses, such as the lack of patient stratification according to histological subtype, among others. Second, in the case of diffuse PTL, our observations and measurements were limited to the anterior–posterior diameter of the thyroid gland, resulting in an inaccurate evaluation of the lesion volume and volume changes post-treatment. Third, CDU was solely used to assess the blood flow grading and was not used to measure spectral parameters such as the resistance index. Furthermore, only one case of CEUS was performed, and the analysis did not include its perfusion parameters or their correlation with PET-CT remission. These limitations underscore the need for larger prospective, multicenter studies to establish the role of ultrasonography in monitoring chemotherapy in patients with PTL.

## 5. Conclusions

Ultrasonography is crucial in the monitoring of and follow-up after chemotherapy in patients with PTL, particularly for early evaluation and repeated dynamic monitoring, and it serves as a valuable complementary method to PET-CT for ongoing patient management. Noteworthy imaging indicators for chemotherapy effectiveness include reduced internal blood flow, a smaller lesion volume, and even the resolution of the lesion. This modality can be tracked and observed at any time, potentially serving as an effective supplementary method to PET-CT. However, the necessity for larger sample studies and technical improvements remains evident. Future research efforts may explore CEUS in the quantitative evaluation of the blood flow perfusion in PTL.

## Figures and Tables

**Table 1:** Patient population and tumor characteristics.

Variables	
Age, years	66.3 ± 20.7 (range: 34–88; median: 67)
Sex	
Female	15
Male	2
Background of Hashimoto’s thyroiditis	
Yes	13
No	4
Clinical stage	
I-II	13
III-IV	4
Clinical manifestation	
Recent significantly enlarged neck mass	13/17
Pain	2
Expiratory dyspnea	2
Systemic symptoms such as fever, etc.	4
Ultrasonography findings (thyroid)	
Diffuse type	5/17
Diffuse lesions involving the unilateral lobe	3
Diffuse lesions involving the bilateral lobes	2
Nodular type	12/17
Total number of nodules	15
Solitary nodule with a clear boundary	9
Solitary nodule with an obscure boundary	1
Multiple nodules with a clear boundary	2
Mixed type	0
Methods of obtaining pathological samples	
CNB	17/17
FNA/EB	0
Pathological type	
DLBCL	13/17
MALToma	1/17
FL	2/17
BL	1/17
Ultrasound (cervical lymph nodes)	
Yes	10/17
No	7/17
Follow-up period, months	30 ± 19 (range: 6–72; median: 24)

DLBCL, diffuse large B-cell lymphoma; MALToma, mucosa-associated lymphatic tissue lymphoma; FL, follicular lymphoma; BL, Burkitt’s lymphoma; CNB, core needle biopsy; FNA, fine needle aspiration; EB, excision biopsy.

**Table 2:** Pretreatment US findings.

US Manifestations	Diffuse Group (*n* = 5)	Nodular Group (*n* = 12, 15 Nodules)
Size on US		
Thyroid gland thickness (cm)	3.73 ± 0.52 (2.7–4.3)	N/A
Maximum diameter (cm)	N/A	4.0 ± 1.8 (1–7.1)
Calculated volume (mL)	N/A	19.8 ± 15.2 (0.19–46.0)
Echogenecity		
Markedly hypoechoic	5/5, 100%	12/15, 80%
Hyper-/iso-/hypoechoic	0/5, 0%	3/15, 20%
“Stripe-shaped” high echoes	5/5, 100%	14/15, 93.3%
Reticulum structures	4/5, 80%	6/15, 40%
Calcification		
Microcalcification	0/5, 0%	0/15, 0%
Macrocalcification	0/5, 0%	1/15, 6.7%
Cystic changes	0/5, 0%	0/15, 0%
Posterior acoustic enhancement		
Present	5/5, 100%	15/15, 100%
Absent	0/5, 0%	0/15, 0%
Adler’s blood flow grading		
Levels 0–1	0/5, 0%	1/15, 6.7%
Levels 2–3	5/5, 100%	14/15, 93.3%
Cervical lymphadenopathy	2/5, 40%	7/12, 41.2%

US, ultrasound; N/A, not applicable.

**Table 3:** Treatment response in the diffuse and nodular types of primary thyroid lymphoma at the first to second, third to fourth, and end cycles of chemotherapy.

Chemotherapy Cycles	Diffuse Group (*n* = 5)				Nodular Group (*n* = 12, 15 Nodules)			
	CR	PR	SD	PD	CR	PR	SD	PD
1–2 cycles	-	5 (100%)	-	-	-	13 (86.7%)	2 (13.3%)	-
3–4 cycles	4 (80%)	1 (20%)	-	-	3 (20%)	8 (53.3%)	2 (13.3%)	2 (13.3%)
End (3–9 cycles)	4 (80%)	-	-	1 (20%)	10 (66.7%)	2 (13.3%)	2 (13.3%)	1 (6.7%)

CR, complete response; PR, partial response; SD, stable disease; PD, disease progression. The effect of the first to second cycles of chemotherapy is based on ultrasound, while the effect of the third to fourth and last cycles is based on PET-CT.

**Table 4:** Thyroid thickness and nodule volume changes after chemotherapy.

Group	Diffuse Group (*n* = 5)	Nodular Group (*n* = 12, 15 Nodules)	
	Maximum Thyroid Gland Thickness	Maximum Nodule Diameter	Nodule Volume
Pre-	3.7 ± 0.5	4.0 ± 1.8	19.8 ± 15.2
Post-	2.1 ± 1.1	1.3 ± 1.9	4.7 ± 9.8
*p*-value	(t = 3.553, *p* = 0.012, 95%CI: 0.5–2.7)	(t = 3.723, *p* = 0.002, 95%CI: 1.1–4.2)	(t = 2.872, *p* = 0.012, 95%CI: 8.7–26.1)

Pre-, prechemotherapy; Post-, postchemotherapy.

**Table 5:** Adler’s semi-quantitative grade changes after chemotherapy.

Group	Diffuse Group (*n* = 5)		Nodular Group (*n* = 12, 15 Nodules)	
	Grades 0–1	Grades 2–3	Grades 0–1	Grades 2–3
Pre-	0	5	1	14
Post-	4	1	12	3

Pre-, prechemotherapy; Post-, postchemotherapy.

## Data Availability

The data that support the findings of this study are available from Peking University Third Hospital, but restrictions apply to the availability of these data, which were used under license for the current study and are thus not publicly available. The data are, however, available from the authors upon reasonable request and with the permission of Peking University Third Hospital.

## Supplementary Materials

The following supporting information can be downloaded at https://www.mdpi.com/article/10.3390/medicina61010015/s1. Figure S1: A 60-year-old woman with multiple follicular lymphomas with a background of Hashimoto’s thyroiditis. (a–f) Ultrasound manifestations before chemotherapy. (a,b) Gray-scale ultrasound revealed multiple hypoechoic nodules (arrows) in the parenchyma of the bilateral lobe, with a maximum diameter of 4.1 cm in the left lobe. (c,d) CDU and micro-blood flow imaging depicting rich and chaotic blood flow signals. (e,f) Multiple enlarged lymph nodes were observed on both sides of the neck, with the largest in left neck region III, measuring approximately 2.4 × 2.0 cm. The cortex was thickened, and some hilar structures were unclear, with grid-like changes inside. CDU: Rich and disordered blood flow signals were seen inside, with a mixed type of blood supply to the hilum (arrow) and edge (arrowheads). (g) PET-CT before chemotherapy showed low-density nodules and masses in both lobes, with significantly increased uptake (arrows) and an SUVmax of 32.0. (h,i) After two cycles of chemotherapy, the ultrasound showed a significant reduction in the lesion size, with only a hypoechoic area of approximately 3.2 × 1.0 cm visible in the left lobe; the remaining nodule lesions had disappeared. (j, k) After five cycles of chemotherapy, the hypoechoic area in the left lobe was reduced to approximately 2.2 × 0.4 cm (arrow), with clear boundaries and a small amount of the punctate blood flow signal inside. (l) PET-CT confirmed no obvious metabolically active lymphoma lesions throughout the body; complete remission was considered. The SUV value was 1.1 in the hypoechoic area of the left lobe, indicating a post-treatment response (arrow). CDU, color Doppler ultrasound; PET-CT, positron emission tomography-computed tomography; SUVmax, maximum standardized uptake value. Figure S2: Kaplan–Meier curves for progression-free survival (a) and overall survival (b) of patients with diffuse vs. nodular types. No significant differences were observed between the two groups.
