# Supplementary material for: Role of Ultrasonography in Monitoring Chemotherapeutic Effects on Primary Thyroid Lymphoma: A Single-Center Retrospective Study
Source: Medicina (Kaunas). 2024 Dec 26;61(1):15. doi: 10.3390/medicina61010015 (PMC11767010; doi:10.3390/medicina61010015)
Supplement: Supplementary file 1 [file medicina-61-00015-s001.zip › medicina-3329835-supplementary.pdf]

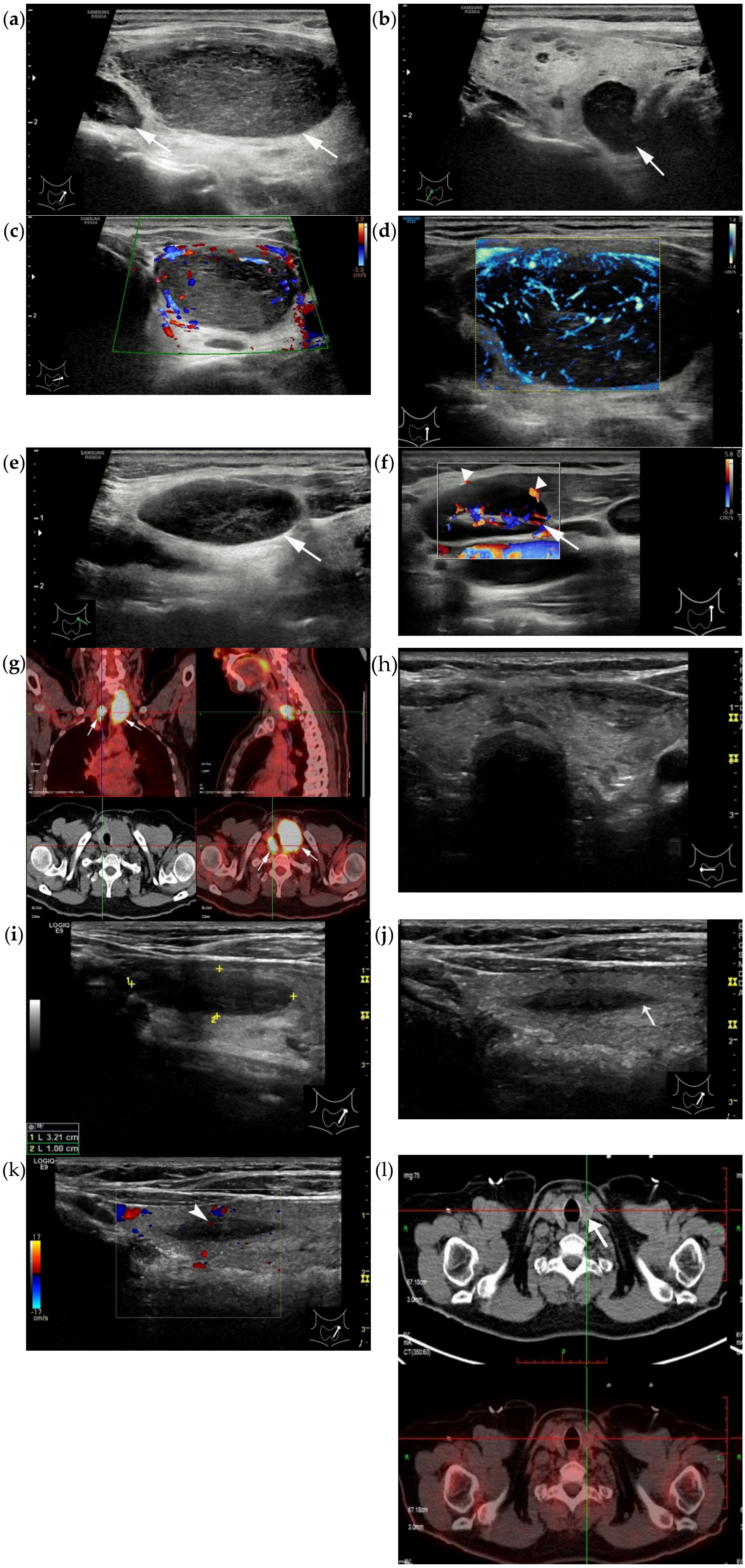
(a)
(b)
(c)
(d)
(e)
(f)
(g)
(h)
(i)
(j)
(k)
(l)
SAMSUNG RS80A
LOGIQ E9
1 L 3.21 cm
2 L 1.00 cm

Figure S1. A 60-year-old woman with multiple follicular lymphomas with a background of Hashimoto's thyroiditis. (a-f) Ultrasound manifestations before chemotherapy. (a, b) Gray-scale ultrasound revealed multiple hypoechoic nodules (arrows) in the parenchyma of the bilateral lobe, with a maximum diameter of 4.1 cm in the left lobe. (c, d) CDU and micro-blood flow imaging depicting rich and chaotic blood flow signals. (e, f) Multiple enlarged lymph nodes were observed on both sides of the neck, with the largest in left neck region III, measuring approximately 2.4 x 2.0 cm. The cortex was thickened, and some hilar structures were unclear, with grid-like changes inside. CDU: Rich and disordered blood flow signals were seen inside, with a mixed type of blood supply to the hilum (arrow) and edge (arrowheads). (g) PET-CT before chemotherapy showed low-density nodules and masses in both lobes, with significantly increased uptake (arrows) and a SUVmax of 32.0. (h, i) After two cycles of chemotherapy, the ultrasound showed a significant reduction in the lesion size, with only a hypoechoic area of approximately 3.2 x 1.0 cm visible in the left lobe; the remaining nodule lesions had disappeared. (j, k) After 5 cycles of chemotherapy, the hypoechoic area in the left lobe was reduced to approximately 2.2 x 0.4 cm (arrow), with clear boundaries and a small amount of the punctate blood flow signal inside. (l) PET-CT confirmed no obvious metabolically active lymphoma lesions throughout the body; complete remission was considered. The SUV value was 1.1 in the hypoechoic area of the left lobe, indicating a post-treatment response (arrow). CDU, color Doppler ultrasound; PET-CT, positron emission tomography–computed tomography; SUVmax, maximum standardized uptake value.

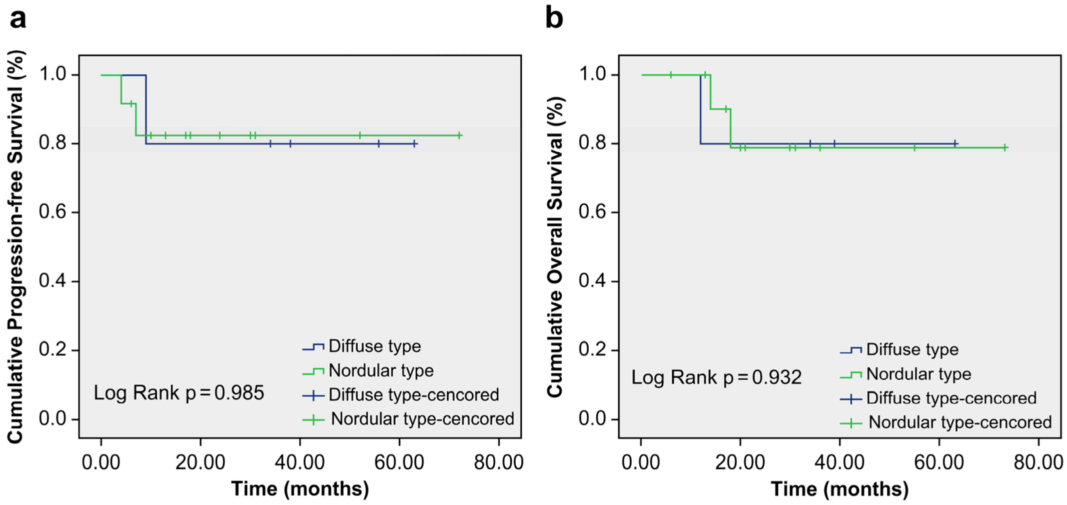

Figure S2. Kaplan–Meier curves for progression-free survival (a) and overall survival (b) of patients with the diffuse vs. nodular type. No significant differences were observed between the two groups.
